# Antarctic Marine Algae Extracts as a Potential Natural Resource to Protect Epithelial Barrier Integrity

**DOI:** 10.3390/md20090562

**Published:** 2022-08-31

**Authors:** Seong-Hee Ko, YoonHee Lim, Eun Jae Kim, Young Wook Ko, In-Sun Hong, Sanghee Kim, YunJae Jung

**Affiliations:** 1 Department of Microbiology, College of Medicine, Gachon University, Incheon 21999, Korea; 2 Lee Gil Ya Cancer and Diabetes Institute, Gachon University, Incheon 21999, Korea; 3 Division of Life Sciences, Korea Polar Research Institute, Incheon 21990, Korea; 4 Department of Molecular Medicine, College of Medicine, Gachon University, Incheon 21999, Korea; 5 Department of Health Science and Technology, Gachon Advanced Institute for Health Science & Technology, Gachon University, Incheon 21999, Korea

**Keywords:** Antarctic marine algae, antioxidant action, barrier organ, epithelium, anti-inflammatory action

## Abstract

The intestine and skin provide crucial protection against the external environment. Strengthening the epithelial barrier function of these organs is critical for maintaining homeostasis against inflammatory stimuli. Recent studies suggest that polar marine algae are a promising bioactive resource because of their adaptation to extreme environments. To investigate the bioactive properties of polar marine algae on epithelial cells of the intestine and skin, we created extracts of the Antarctic macroalgae *Himantothallus grandifolius*, *Plocamium cartilagineum*, *Phaeurus antarcticus*, and *Kallymenia antarctica*, analyzed the compound profiles of the extracts using gas chromatography-mass spectrometry, and tested the protective activities of the extracts on human intestinal and keratinocyte cell lines by measuring cell viability and reactive oxygen species scavenging. In addition, we assessed immune responses modulated by the extracts by real-time polymerase chain reaction, and we monitored the barrier-protective activities of the extracts on intestinal and keratinocyte cell lines by measuring transepithelial electrical resistance and fluorescence-labeled dextran flux, respectively. We identified bioactive compounds, including several fatty acids and lipid compounds, in the extracts, and found that the extracts perform antioxidant activities that remove intracellular reactive oxygen species and scavenge specific radicals. Furthermore, the Antarctic marine algae extracts increased cell viability, protected cells against inflammatory stimulation, and increased the barrier integrity of cells damaged by lipopolysaccharide or ultraviolet radiation. These results suggest that Antarctic marine algae have optimized their composition for polar environments, and furthermore, that the bioactive properties of compounds produced by Antarctic marine algae can potentially be used to develop therapeutics to promote the protective barrier function of the intestine and skin.

## 1. Introduction

The intestine and skin are crucial interface organs that communicate with the exogenous environment [1]. Epithelial cells covering the intestine and skin serve as the first line of defense against invading microorganisms while maintaining important connections between the internal body and the external environment [2]. The epithelial cells of the intestine constitute a physiological barrier and perform immunological functions, such as sensing and sampling antigens, secreting antimicrobial molecules, and shaping and guiding mucosal immune responses [3]. Disruption of this barrier can lead to conditions such as noxious inflammation and destruction of the digestive tract [4]. Similarly, the epithelial cells of the skin act as a barrier to protect internal organs from external threats, such as ultraviolet (UV) radiation [5]. It is well known that UVB radiation comes into direct contact with the stratified squamous epithelium [6]. As a result, skin epithelial cells are continuously exposed to oxidative stress from reactive oxygen species (ROS) caused by UVB exposure [7]. The common anatomical properties and barrier functions of the skin and intestine mean that gastrointestinal health and skin homeostasis are interconnected [1,8]. It is essential to strengthen the barrier function of epithelial cells of the intestine and skin to protect the body from the external environment, oxidative stress, and inflammatory substances. Despite a vast literature addressing pathological conditions associated with epithelial dysfunction of the intestine and skin, such as atopic dermatitis and inflammatory bowel diseases [9,10,11,12], current strategies for the protection or repair of the epithelial barrier are limited. 

The Arctic and Antarctic polar circles, where temperatures are typically below 5 °C, occupy 14% of the total biosphere [13]. Although it was once generally accepted that life becomes static at low temperatures, the metabolism and natural production of polar organisms are not stopped by the cold conditions of the polar circles [14]. To sustain growth and metabolic activity in sub-freezing temperatures and extreme environments characterized by complete darkness in winter and constant light and high UV radiation exposure in summer [15,16], polar organisms have acquired many metabolic, structural, and functional adaptations [13]. As a result, natural products synthesized by polar organisms may provide advantages in terms of protection against harsh environments compared with products synthesized by organisms in less extreme environments. Exploration of polar resources may, therefore, lead to the identification of novel natural products with bioactivity promoting protective barrier integrity. Marine algae are among the most promising sources of novel bioactive compounds, displaying antioxidant, antibacterial, antifungal, antidiabetic, and anti-inflammatory activities [17]. A recent study reported that bioactive compounds isolated from Antarctic macroalgae have novel antimicrobial and anticancer properties [18]. It is still unknown, however, whether polar marine algae have protective bioactivities against inflammatory stimuli that induce tissue damage.

We collected extracts of four Antarctic marine algae: *Himantothallus grandifolius*, *Plocamium cartilagineum*, *Phaeurus antarcticus*, and *Kallymenia* *antarctica*. We then analyzed compounds in the extracts using gas chromatography-mass spectrometry (GC–MS) and performed assays to evaluate the ability of the extracts to protect skin and intestinal epithelial cells against inflammatory or radiation-induced damage. We evaluated the protective effects of the Antarctic marine algae extracts against lipopolysaccharide (LPS)-induced and UVB-induced damages in human intestinal epithelial Caco-2 cells and human keratinocyte HaCaT cells, respectively. We found that the Antarctic marine algae extracts have fatty acid and lipid-rich compositional features of adaptation to the extreme polar environment and exert scavenging activity against specific radicals and intracellular ROS. We also found that the Antarctic marine algae extracts protect the barrier integrity of Caco-2 and HaCaT cells against disruption caused by exposure to LPS and UVB, respectively. These findings suggest that Antarctic marine algae are a potential resource for compounds that promote barrier function and help maintain homeostasis of the intestine and skin. 

## 2. Results

### 2.1. GC–MS Profiles 

**Figure 1:**
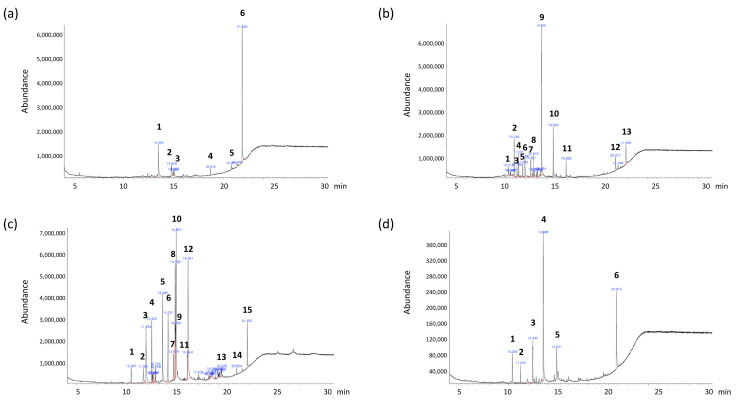
Chromatographic profiles of the polar marine algae extracts. (**a**) *Himantothallus grandifolius,* (**b**) *Plocamium cartilagineum*, (**c**) *Phaeurus antrcticus*, and (**d**) *Kallymenia Antarctica*. The identities of the compounds are shown in Table 1, Table 2, Table 3 and Table 4.

We performed GC–MS analysis to identify compounds present in the polar marine algae extracts. Chromatographic profiles revealed six, thirteen, fifteen, and six compounds in extracts of *H. grandifolius*, *P. cartilagineum*, *P. antarcticus*, and *K.* *antarctica*, respectively (representative chromatographic profiles shown in Figure 1). Among these compounds were several fatty acids. Extracts of all four polar marine algae species contained n-Hexadecanoic acid (palmitic acid). *H. grandifolius* and *K. antarctica* extracts also contained 9-Octadecenoic acid (oleic acid). The other fatty acids identified in the extracts were tetradecanoic acid (myristic acid) in *P. cartilagineum* extracts and octadecanoic acid (stearic acid) and 9,12-octadecadienoic acid (linoleic acid) in *H. grandifolius* extract and *P. antarcticus* extract, respectively. In addition to the fatty acids, cholesterol was found in *K. antarctica* extract, and the essential fat-soluble vitamin E was detected in *H. grandifolius* and *P. antarcticus* extracts. Other bioactive compounds in the extracts included the anti-inflammatory compound neophytadiene [19] in *P. cartilagineum* extracts and the antioxidant and antibacterial compound heptadecane [20] in *P. cartilagineum* and *K.* *antarctica* extracts. We next performed further experiments to investigate the biological potential of the various bioactive compounds in the polar marine algae extracts.

### 2.2. Radical-Scavenging Activity

*Spirulina* sp. is a microscopic filamentous cyanobacterium with a long history as a safe, functional food for humans [21]. Therefore, we used *Spirulina* collected from a non-polar area as a positive control for our studies of Antarctic marine algae.

**Figure 2:**
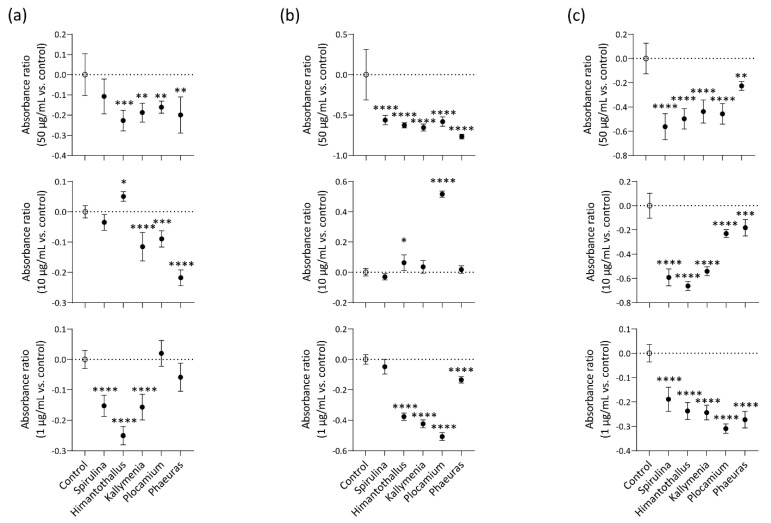
Radical-scavenging activity of the marine algae extracts. (**a**) Hydrogen peroxide scavenging activity. The marine algae extracts were incubated with dichlorohydrofluorescein (DCHF)-DA and H_2_O_2_; (**b**) Hydroxyl radical-scavenging activity. The marine algae extracts were incubated with DCHF-DA, H_2_O_2_, and FeCl_2_; (**c**) Peroxynitrite scavenging activity of marine algae. The marine algae extracts were incubated with DCHF-DA and ONOO-. *n* = 5/group. The absorbance ratio was calculated by comparing the fluorescence of the marine algae-treated group to that of the untreated control. Data are presented as the mean ± SD. *P* values are determined by one-way ANOVA with Turkey’s multiple comparisons test. * *p* < 0.05, ** *p* < 0.01, *** *p* < 0.001, and **** *p* < 0.0001 vs. Control.

First, we assessed specific radical-scavenging activities of marine algae extracts reconstituted at 50 μg/mL, 10 μg/mL, and 1 μg/mL by measuring their abilities to inhibit 2′,7′-dichlorofluorescein diacetate (DCHF)-DA fluorescence induced by hydrogen peroxide (H_2_O_2_), hydroxyl radical (OH^.^), or peroxynitrite (ONOO-). At 50 μg/mL, all four Antarctic marine algae extracts inhibited fluorescence induced by H_2_O_2_ (Figure 2a), OH^.^ (Figure 2b), or ONOO- (Figure 2c) in comparison with a negative control containing only DCHF-DA and H_2_O_2_, OH^.^, or ONOO-. There was no significant difference in H_2_O_2_-induced fluorescence between the negative control and the positive-control *Spirulina* extract at 50 μg/mL (*p* = 0.1048; Figure 2a). At 10 μg/mL, the *Kallymenia*, *Plocamium*, and *Phaeurus* extracts inhibited H_2_O_2_-induced fluorescence (Figure 2a), and all of the marine algae extracts inhibited fluorescence induced by ONOO- (Figure 2c); however, the *Himantothallus* and *Plocamium* extracts at 10 μg/mL increased fluorescence induced by OH^.^ (Figure 2b). At 1 μg/mL, the *Spirulina*, *Himantothallus* and *Kallymenia* extracts inhibited H_2_O_2_-induced fluorescence (Figure 2a), and all four Antarctic marine algae extracts inhibited fluorescence induced by OH^.^ or ONOO- (Figure 2b,c).

### 2.3. Cell viability Protection

**Figure 3:**
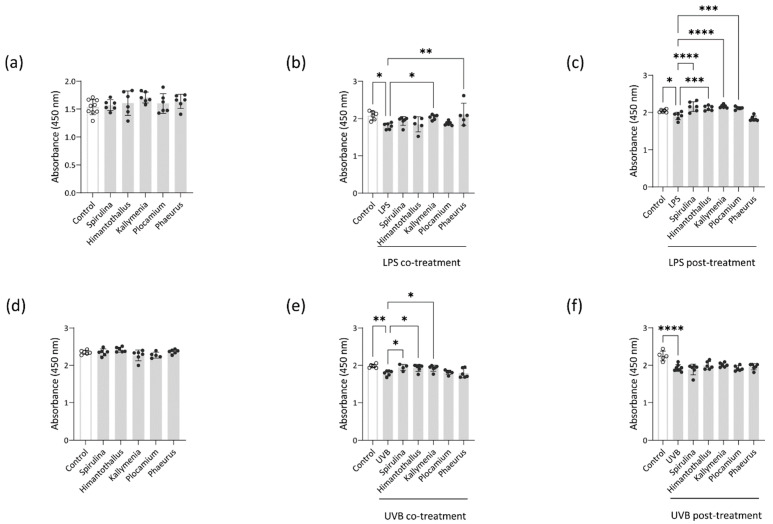
Effects of marine algae extract on the viability of Caco-2 cells and HaCaT cells. (**a**) Viability of Caco-2 cells treated with the indicated marine algae extracts for 18 h; (**b**) Viability of Caco-2 cells treated with the indicated marine algae extracts and LPS for 18 h; (**c**) Viability of Caco-2 cells treated with LPS for 18 h after overnight pretreatment with the indicated marine algae extracts; (**d**) Viability of HaCaT cells treated with the indicated marine algae extracts for 18 h; (**e**) Viability of HaCaT cells irradiated with ultraviolet (UV)B and incubated in the presence of indicated marine algae extracts for 18 h; (**f**) Viability of HaCaT cells that were pretreated overnight with the indicated marine algae extracts, irradiated with UVB, and incubated for 18 h. Data are presented as the mean ± SD. *p* values are determined by one-way ANOVA with Turkey’s multiple comparisons test. * *p* < 0.05, ** *p* < 0.01, *** *p* < 0.001, and **** *p* < 0.0001.

To determine the cytotoxicity of the *H. grandifolius*, *P. cartilagineum*, *P. antarcticus*, and *K. antarctica* extracts to Caco-2 cells and HaCaT cells, we incubated each cell line for 18 h with each extract. Although some extracts decreased the viability of the cells at 10 μg/mL (data not shown), the viability of Caco-2 and HaCaT cells was unaltered by treatment with any of the Antarctic marine algae extracts at 1 μg/mL, indicating no apparent toxicity to intestine and skin epithelial cells within a specific range of concentration (Figure 3a,d). To assess whether the Antarctic marine algae extracts could protect Caco-2 and HaCaT cells against damaging stimuli, we measured the viability of LPS-treated Caco-2 cells and UVB-treated HaCaT cells in the presence of the extracts. We selected 18 h of treatment of Caco-2 cells with 1 μg/mL LPS and 4 h of culture of HaCaT cells after exposure to 25 mJ/cm^2^ UVB radiation as the optimal damaging conditions (Figure S1). As shown in Figure 3b, co-treatment with 1 μg/mL *Kallymenia* or *Phaeurus* extract protected Caco-2 cells from LPS-induced toxicity. Similarly, overnight pretreatment with *Spirulina*, *Himantothallus*, *Kallymenia*, or *Plocamium* extract at 1 μg/mL prior to LPS exposure also protected the Caco-2 cells from LPS-induced toxicity (Figure 3c). On the other hand, co-treatment with 1 μg/mL *Spirulina* extract had no significant effect on LPS-induced toxicity in Caco-2 cells (*p* = 0.4378; Figure 3b). In UVB-exposed HaCaT cells, co-treatment with 1 μg/mL of *Spirulina*, *Himantothallus*, or *Kallymenia* extract significantly increased viability compared with that in cells exposed to UVB without any co-treatment (Figure 3e). Neither *Spirulina* extract nor any of the Antarctic marine algae extracts improved the viability of UVB-irradiated HaCaT cells when the cells were pretreated with the extracts overnight prior to UVB exposure (Figure 3f). These findings indicate that extracts of Antarctic marine algae administered at a concentration of 1 μg/mL have protective effects against inflammatory and radiation-induced damage to epithelial cells without inducing cytotoxicity in vitro.

### 2.4. Intracellular ROS Scavenging

**Figure 4:**
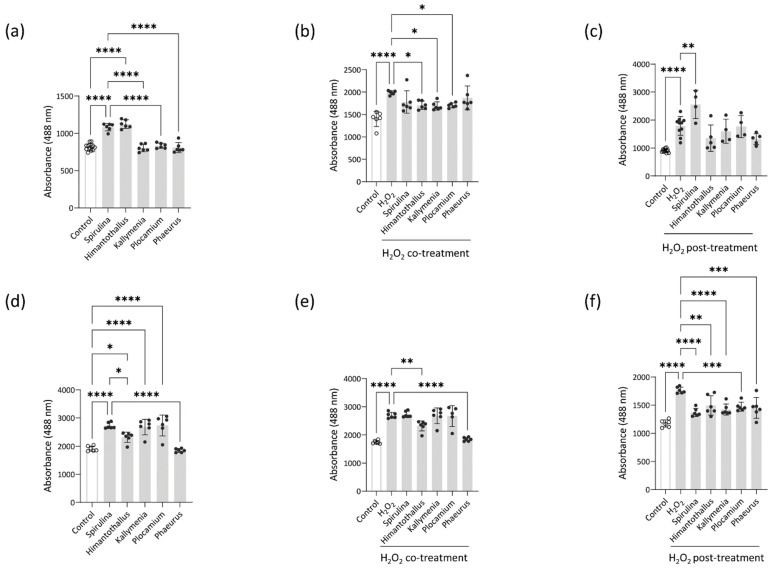
Intracellular reactive oxygen species (ROS) scavenging activity of the marine algae extracts on Caco-2 cells and HaCaT cells. (**a**) Oxidation of Caco-2 cells treated with the indicated marine algae extracts for 18 h; (**b**) Oxidation of Caco-2 cells treated for 18 h with H_2_O_2_ and the indicated marine algae extracts; (**c**) Oxidation of Caco-2 cells treated with H_2_O_2_ for 18 h after overnight pretreatment with the indicated marine algae extracts; (**d**) Oxidation of HaCaT cells treated with the indicated marine algae extracts for 18 h; (**e**) Oxidation of HaCaT cells treated for 18 h with H_2_O_2_ and the indicated marine algae extracts; (**f**) Oxidation of HaCaT cells that were treated with H_2_O_2_ for 18 h after overnight pretreatment with the indicated marine algae extracts. Data are presented as the mean ± SD. *P* values are determined by one-way ANOVA with Turkey’s multiple comparisons test. * *p* < 0.05, ** *p* < 0.01, *** *p* < 0.001, and **** *p* < 0.0001.

Oxidative stress occurs when ROS generation overcomes cells’ antioxidant defenses, which leads to oxidative damage to nucleic acids, proteins, and lipids [22]. In the absence of any other stimuli, Caco-2 cells treated with 1 μg/mL *Spirulina* or *Himantothallus* extract, and HaCaT cells treated with *Spirulina*, *Himantothallus*, *Kallymenia*, or *Plocamium* extracts at 1 μg/mL, showed a significant increase of ROS levels (Figure 4a,d). In contrast, Caco-2 cells treated with *Kallymenia*, *Plocamium*, or *Phaeurus* extracts had lower ROS levels than Caco-2 cells treated with *Spirulina* extract, and HaCaT cells treated with *Himantothallus* or *Phaeurus* extracts also showed lower ROS levels than HaCaT cells treated with *Spirulina* extract (Figure 4a,d).

We next explored whether the Antarctic marine algae extracts could protect Caco-2 and HaCaT cells against acute oxidative stress induced by H_2_O_2_. We first exposed Caco-2 and HaCaT cells to 250 mM, 500 mM, and 1000 mM H_2_O_2_ for 18 h. As a significant increase of ROS was detected at all three concentrations of H_2_O_2_ (Figure S2), we used the lowest concentration of 250 mM H_2_O_2_ as an oxidative stimulus for the cells. As shown in Figure 4b, co-treatment with *Himantothallus*, *Kallymenia*, or *Plocamium* extract significantly reduced H_2_O_2_-induced ROS levels in Caco-2 cells. Overnight pretreatment with any of the Antarctic marine algae extracts similarly resulted in decreased ROS levels in H_2_O_2_-stimulated Caco-2 cells, whereas pretreatment with *Spirulina* extract resulted in significantly increased ROS levels compared with that in H_2_O_2_-exposed Caco-2 cells without any pretreatment (Figure 4c). In HaCaT cells, co-treatment with *Himantothallus* or *Phaeurus* extract reduced H_2_O_2_-induced ROS levels (Figure 4e), and overnight pretreatment with *Spirulina* extract or any of the Antarctic marine algae extracts reduced H_2_O_2_-induced ROS levels (Figure 4f). Overall, these results indicate that although some marine algae extracts induced ROS production in intestine and skin epithelial cells within a specific concentration range, the Antarctic marine algae extracts suppressed further H_2_O_2_-induced ROS production, especially when the cells were treated with the extracts in advance of H_2_O_2_ exposure.

### 2.5. Anti-Inflammatory Activity

**Figure 5:**
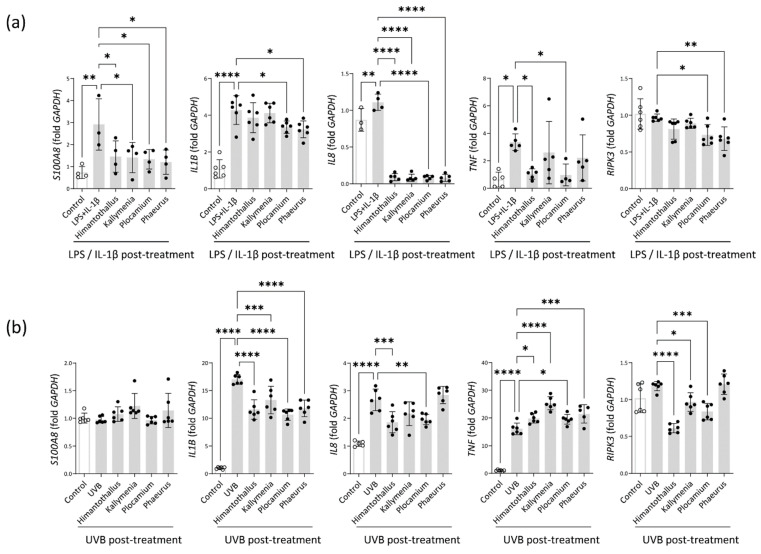
Effects of the Antarctic marine algae extracts on the expression of inflammatory markers in Caco-2 cells and HaCaT cells. (**a**) mRNA expression of *S100A8*, *IL1B*, *IL8*, *TNF*, and *RIPK3* in Caco-2 cells treated with LPS and IL-1β after overnight incubation with the indicated Antarctic marine algae extracts; (**b**) mRNA expression of *S100A8*, *IL1B*, *IL8*, *TNF*, and *RIPK3* in UVB-irradiated HaCaT cells cultured for 4 h. The HaCaT cells were pretreated with the indicated Antarctic marine algae extracts for 18 h before UVB irradiation. Data are presented as the mean ± SD. *p* values are determined by one-way ANOVA with Turkey’s multiple comparisons test. * *p* < 0.05, ** *p* < 0.01, *** *p* < 0.001, and **** *p* < 0.0001.

Epithelial permeability is modulated by inflammation [23]. Although LPS alone was incapable of inducing mRNA expression of IL-1β, a cytokine involved in both the initiation and the amplification of the inflammatory response leading to intestinal injury [24], the combination of LPS and 1 μg/mL IL-1β induced significant upregulation of *IL1B* and *TNF* in Caco-2 cells (*IL1B*, *p* = 0.0003; *TNF*, *p* < 0.0001; Figure S3A). Next, we assessed whether pretreatment with the Antarctic algae extracts could inhibit the inflammatory responses of Caco-2 cells induced by LPS and IL-1β. As shown in Figure 5a, treatment with LPS and IL-1β upregulated mRNA expression of *S100A8*, *IL1B*, *IL8*, and *TNF* in Caco-2 cells. Pretreatment with any of the four Antarctic algae extracts significantly inhibited the upregulation of *S100A8* and *IL8* induced by the LPS and IL-1β treatment. Similarly, pretreatment with *Plocamium* or *Phaeurus* extract inhibited the upregulation of *IL1B*, and pretreatment with *Himantothallus* or *Plocamium* extract inhibited the upregulation of *TNF* (Figure 5a).

When HaCaT cells were exposed to 25 mJ/cm^2^ UVB radiation, *IL1B*, *IL8*, and *TNF* mRNA expression was significantly upregulated (Figure S3B and Figure 5b). All four Antarctic algae extracts significantly reduced the UVB-induced upregulation of *IL1B* and *TNF*, and the *Himantothallus* and *Plocamium* extracts significantly reduced the upregulation of *IL8* (Figure 5b). Activation of receptor interacting protein kinase 3 (*RIPK3*) is associated with necrotic cell death after exposure to excessive inflammatory stress [25]. Although *RIPK3* expression was not significantly different between untreated Caco-2 control cells and cells treated with LPS and IL-1β (*p* = 0.9490), *RIPK3* expression was significantly lower in cells that were pretreated with *Plocamium* or *Phaeurus* extract before LPS and IL-1β exposure (Figure 5a). Similarly, *RIPK3* expression was lower in HaCaT cells that were pretreated with *Himantothallus*, *Kallymenia*, or *Plocamium* extract prior to UVB exposure than in cells that were exposed to UVB with no pretreatment (Figure 5b).

### 2.6. Epithelial Barrier Protection

**Figure 6:**
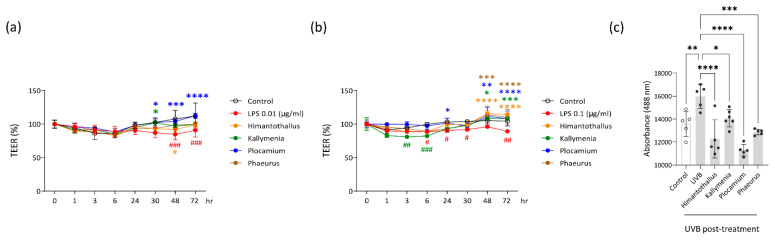
Effects of the marine algae extracts on the permeability of Caco-2 cells and HaCaT cells. (**a**) Transepithelial electrical resistance (TEER) changes of polarized Caco-2 cells treated with 0.01 μg/mL LPS after overnight pretreatment with the indicated Antarctic marine algae extracts; (**b**) TEER changes of polarized Caco-2 cells treated with 0.1 μg/mL LPS after overnight pretreatment with the indicated Antarctic marine algae extracts; (**c**) Fluorescence of 4-kDa fluorescein isothiocyanate (FITC)-dextran fluxed from the apical to the basal compartment of transwells seeded with HaCaT cells. Before FITC-dextran addition, the HaCaT cells were irradiated with UVB (25 mJ/cm^2^) after overnight pretreatment with the indicated marine algae extracts. Data are presented as the mean ± SD. *p* values are determined by two-way ANOVA with Bonferroni’s multiple comparisons test (a and b) or one-way ANOVA with Turkey’s multiple comparisons test (c). * *p* < 0.05, ** *p* < 0.01, *** *p* < 0.001, and **** *p* < 0.0001 vs. LPS. # *p* < 0.05, ## *p* < 0.01, and ### *p* < 0.001 vs. Control. The color of * and # corresponds to the color of the treatment indicated in the graph.

To determine whether the Antarctic marine algae extracts could help maintain the barrier function of skin and intestine epithelial cells, we analyzed the permeability of Caco-2 cells by measuring transepithelial electrical resistance (TEER). The TEER of Caco-2 cells was increased once the cells formed a monolayer of polarized cells (Figure S4). The TEER of the polarized Caco-2 cells was significantly decreased, consistent with increased permeability, after 48 h or 72 h treatment with 0.01 μg/mL LPS and after 6 h, 24 h, 30 h, or 72 h treatment with 0.1 μg/mL LPS (Figure 6a,b). Co-treatment with *Plocamium* extract reversed the 0.01 μg/mL LPS-induced permeability at 30 h, 48 h, and 72 h and the 0.1 μg/mL LPS-induced permeability at 24 h, 48 h, and 72 h (Figure 6a,b). *Kallymenia* extract showed protective effects against 0.01 μg/mL LPS-induced permeability at 30 h and against 0.1 μg/mL LPS-induced permeability at 48 h and 72 h (Figure 6a,b). *Himantothallus* and *Phaeurus* extracts both reversed 0.1 μg/mL LPS-induced permeability at 48 h and 72 h (Figure 6b). We could not observe spontaneous TEER increase in HaCaT cells (Figure S4), so we used fluorescein isothiocyanate (FITC)-labeled dextran flux to assess the barrier integrity of those cells. As shown in Figure 6c, pretreatment of HaCaT cells with *Himantothallus*, *Kallymenia*, *Plocamium*, or *Phaeurus* extracts in advance of UVB exposure significantly reduced the permeable flow of FITC-dextran.

## 3. Discussion

We demonstrated that four kinds of Antarctic marine algae (*H. grandifolius*, *P. cartilagineum*, *P. antarcticus*, and *K. antarctica*) possess a number of bioactive compounds, scavenge specific radicals, increase cell viability, and protect the barrier integrity of human intestinal epithelial and keratinocyte cell lines exposed to LPS-induced or UVB-induced inflammatory damage. 

Hydrogen peroxide causes oxidative stress and can cross cell membranes quickly, and once inside the cell, it can react with iron to form even more harmful species, such as hydroxyl radicals [26]. Hydroxyl radicals form 8-hydroxy-guanine adducts, which cause point mutations as well as single-strand and double-strand DNA breaks [27]. Extracts of all four Antarctic marine algae at concentrations of 50 μg/mL scavenged H_2_O_2_ and OH^.^. Although ONOO- is non-radical, its ionized form is more reactive than its molecular form, causing protein denaturation and lipid peroxidation, destroying DNA, and reacting with thiols [28]. Because no enzyme specifically removes ONOO- in the human body, it is important to use endogenous materials and natural and synthetic chemicals to scavenge ONOO- to prevent toxicity [29]. Importantly, all four Antarctic marine algae extracts demonstrated scavenging ability on ONOO- under all tested concentrations.

After confirming that 1 μg/mL of Antarctic marine algae extracts improved radical-scavenging without reducing the viability of Caco-2 and HaCaT cells, we assessed the bioactivities of the four Antarctic marine algae extracts using cells treated with LPS or UVB radiation. Pretreatment or co-treatment with the extracts increased viability and inhibited H_2_O_2_ production in LPS-stimulated Caco-2 cells, suggesting that Antarctic marine algae produce compounds that might prevent inflammation-associated intestinal epithelial damage. *Himantothallus* and *Kallymenia* extracts protected HaCaT cells against cytotoxic UVB exposure. Considering that Antarctic marine algae are naturally adapted to survive high levels of UVB exposure in polar regions, as most are immobile and unable to avoid the radiation [16], identification of a UV-absorbing substance in the Antarctic marine algae might reveal the mechanisms underlying the protection afforded to the HaCaT cells.

The intestinal epithelium is constantly exposed to ROS inducers, such as commensal microorganisms and food antigens [30]. Likewise, the skin is a major target tissue for oxidative stress from ROS that originates in the environment [31]. Uncontrolled production or inadequate removal of ROS accelerates inflammatory responses, as demonstrated by intestinal damage in mice in the absence of ROS regulators, and by increased production of proinflammatory cytokines in ROS-stimulated keratinocytes [30,32]. Therefore, we focused on the potential of Antarctic marine algae to inhibit ROS production in Caco-2 intestinal epithelial cells and HaCaT keratinocytes. Co-treatment of H_2_O_2_-exposed Caco-2 cells with *Himantothallus*, *Kallymenia*, or *Plocamium* extract inhibited H_2_O_2_-induced ROS production. The *Himantothallus*, *Kallymenia*, and *Plocamium* extracts also suppressed expression of *S100A8* and *IL8* in Caco-2 cells stimulated with LPS and IL-1β. In HaCaT cells, co-treatment with *Himantothallus* or *Phaeurus* extracts inhibited H_2_O_2_-induced ROS production and UVB-induced expression of *IL1B* and *TNF*. Therefore, it is plausible to suggest that Antarctic marine algae can play a role in blocking crosstalk between ROS and proinflammatory cytokines during the inflammation process. This idea is further supported by the GC–MS profiles of the Antarctic marine algae. The fatty acid n-Hexadecanoic acid, which was found in all four marine algae extracts, is known to inhibit phospholipase A2, an enzyme that initiates inflammation by releasing fatty acids from membrane phospholipids [33]. Another fatty acid, 9-Octadecenoic acid, was found in the *H. grandifolius* and *K. antarctica* extracts and is widely known to have anti-inflammatory activity [34]. The *H. grandifolius* and *P. antarcticus* extracts also contained Vitamin E, which has been studied for its antioxidant properties and protective effects against dementia, aging, and cancer [35]. In addition, 3-aminobenzamide and neophytadiene were detected in *P. cartilagineum* extract and were previously shown to alleviate DNA damage [36] and to have potent anti-inflammatory and antimicrobial activities [37], respectively. Only the *P. cartilagineum* extract inhibited the expression of all the tested inflammatory mediators in Caco-2 cells stimulated with LPS and IL-1β, implying that compounds present in *P. cartilagineum*, but not the other polar algae species, play a pivotal role in protecting intestinal epithelial cells against inflammatory responses. 

Pretreatment with any of the Antarctic marine algae extracts significantly decreased H_2_O_2_-induced ROS production in HaCaT cells, implying a preventive role against oxidative stress-induced skin damage. It was previously reported that a cream containing the microalgae pigment Fucoxanthin prevented epidermal hyperplasia and UVB-induced skin erythema in mice [38]. The bioactivities of Antarctic marine algae extracts in reducing ROS and proinflammatory cytokine levels in HaCaT cells suggest that Antarctic marine algae might contain useful compounds for topical applications to control UVB-induced skin inflammation. 

The extensive surface area of the intestine and skin covered by epithelium provides the first line of defense against harmful external stimuli [1]. As the intestine and skin are populated with commensal bacteria, their epithelial barrier function is critical for maintaining tissue integrity while defending against pathogens [39]. Our results suggest that Antarctic marine algae produce compounds that protect against disruption of the barrier integrity of Caco-2 cells and HaCaT cells. The intestine and skin each have tissue-specific physical, microbial, and immunologic barriers [39]. All four Antarctic marine algae extracts showed protective activity against barrier breakdown of Caco-2 cells and HaCaT cells, as demonstrated by a decrease of TEER and FITC-dextran flux after exposure to LPS and UVB, respectively. All of the Antarctic marine algae extracts contained several lipid compounds and fatty acids such as palmitic acid, oleic acid, myristic acid, stearic acid, and linoleic acid, which play a role in epithelial barrier protection. Fatty acids and cholesterol promote barrier preservation in the skin epithelium, providing protection against pathologic skin conditions [40]. In the intestine, the addition of fatty acid to intestinal mucin is important for maintaining gut barrier function [41]. Moreover, innate immune cells in the tissues use fatty acids to maintain barrier immunity [42]. The abundance of fatty acids and lipid compounds in the polar marine algae extracts might be indicative of adaptations to optimize the compositional features of the algae for the extreme polar environment. We further suggest that the fatty acid-rich properties of Antarctic marine algae contribute to the barrier-protective activities of their extracts. Considering the pivotal role of commensal microbiota in maintaining epithelial integrity and tissue homeostasis of the intestine and skin [43], further studies are required to investigate the influence of Antarctic marine algae extracts on the microbiota of these tissues. 

Because of its high polarity, methanol produces a high extraction yield and extracts both lipophilic and hydrophilic molecules [44]. Although the toxic characteristic of methanol should be considered, some studies addressed the use of methanol extracts without significant toxicity [44,45,46]. We employed methanol extraction to create the Antarctic marine algae extracts. Methanol-based extracts of the Antarctic marine algae within a specific concentration range were nontoxic for cell proliferation and showed protective activity in Caco-2 and HaCaT cells in response to inflammatory and radiation insults. Considering that the bioactivity of the marine algae extracts might be influenced by extraction protocols [18], other solvents should be tested in the future to optimize the extraction. Ethanol is a good candidate as it is effective in separating bioactive compounds from plant materials and is safe for human consumption [47].

We did not attempt to identify specific bioactivities of the individual compounds detected in the polar marine algae extracts; however, we demonstrated the presence of a number of fatty acids and lipid mediators with known bioactivities. Our findings are in line with previous studies suggesting that lipids from macroalgae can be a natural source of fatty acids with potential for pharmaceutical application [48,49]. Moreover, by demonstrating the protective effects of the extracts on damaged Caco-2 and HaCaT cells, we provided additional evidence that marine algae from the Antarctic region are a promising source of substances with radical-scavenging, anti-inflammatory, and barrier-protective activities. Further research should be done to explore bioactive compounds from the Antarctic marine algae used in this study and to potentially enable their future application as therapeutic tools.

## 4. Materials and Methods

### 4.1. Sample Collection

*H. grandifolius*, *P. cartilagineum*, *P. antarcticus*, and *K.antarctica* were obtained from the Antarctic region between December and February in 2018 or 2019. The location and year of each sample collection were as follows: *H. grandifolius* (S62°13’19.20" W58°47’16.80", 2019), *P. cartilagineum* (S62°13’19.20" W58°47’16.80", 2018), *P. antarcticus* (S 62°12’39.25" W58°54’55.21", 2019), and *K. antarctica* (S62°14’21.96" W58°46’36.27", 2019). Each sample was deposited at the Korea Polar Research Institute, Republic of Korea.

### 4.2. Sample Preparation

Ten grams of dried marine algae material were placed in a 50 mL conical tube, and 40 mL of 80% methanol in grade 3 distilled water was added. The conical tubes were kept in a reciprocating shaker for 24 h at room temperature and then filtered through Whatman no. 1 filter paper. The solvent was removed from the extract using a rotary vacuum evaporator with a water bath temperature of 37 °C. The collected residue was then dissolved in dimethyl sulfoxide. 

### 4.3. GC–MS Analysis

GC–MS analysis was performed using an Agilent 7890 B gas chromatograph equipped with a 5977B mass selective detector (GC/MSD) (Agilent Technologies, Santa Clara, CA, USA). Chromatographic separation was achieved using a Rxi-5HT capillary column (30 m × 0.25 mm I.D., 0.25 μm film thickness; RESTEK, Bellefonte, PA, USA). An automatic autosampler (Agilent 7683B) was used for all experiments. The temperature of the injector was 280 °C. One microliter of each extract was injected in split mode with a ratio of 1/50. The carrier gas was helium C-60 at a constant flow of 1 mL/min. The GC oven temperature was initially 60 °C for 1 min, increased to 160 °C in 3 °C/min increments, and then increased to 320 °C in 10 °C/min increments, followed by a hold for 10 min. The mass spectrometer was tuned on electron ionization mode at 70 eV with the ion source temperature set at 250 °C. The running time was 30 min. Scan mode was used in the range of 30–600 m/z with a scan rate of 2.6 scan/s. The Agilent Mass Hunter Qualitative Analysis B.04.00 software was used for data analysis. Single compounds were identified by comparing mass spectra with NIST mass spectral libraries (National Institute of Standards, 2005 version) and the Wiley Registry 8th Edition.

### 4.4. Cell Culture

Caco-2 cell line was purchased from the Korean cell line bank (KCLB, Seoul, Korea) and HaCaT cell line provided by Dr. Eui-Ju Yeo (Gachon University, Incheon, Korea). Caco-2 cells and HaCaT cells were cultured at 37 °C in a 5% CO_2_ atmosphere in modified Eagle medium (MEM) and Dulbecco′s Modified Eagle′s Medium (DMEM)-high glucose, respectively, supplemented with 10% (*v*/*v*) fetal bovine serum, 1% (*v*/*v*) penicillin-streptomycin, 1 M hydroxyethyl piperazineethanesulfonic acid, 100 mM sodium pyruvate, 50 mM 2-mercaptoethanol, 10 mM non-essential amino acid, and L-Glutamine. For inflammatory stimuli, Caco-2 cells were treated with 1 μg/mL LPS (Sigma–Aldrich, St. Louis, MO, USA) with or without 1 μg/mL IL-1β (PeproTech, Rocky Hill, NJ, USA). HaCaT cells were irradiated with 25 mJ/cm^2^ UVB and then cultured for 4 h to induce epithelial damage.

### 4.5. Radical-Scavenging Assay

DCHF-DA 50 μM (Sigma–Aldrich) and algae extract were incubated with 10 mM H_2_O_2_ (Sigma–Aldrich), 60 mM H_2_O_2,_ and 0.75 mM FeCl_2_, or 0.5 mM ONOO- in pH 7.4 phosphate-buffered saline (PBS) at 37 °C for 10 min. All experiments were performed in a dark room to prevent oxidation of the DCHF-DA. Fluorescence quenching was measured using a spectrofluorometer (VICTOR3, PerkinElmer, Waltham, MA, USA) with excitation and emission wavelengths of 488 nm and 530 nm, respectively.

### 4.6. Cell Viability Assay

The effect of algae extracts on the viability of Caco-2 and HaCaT cells was determined using a water-soluble tetrazolium salt assay kit according to the manufacturer’s instructions (EZ-Cytox cell viability assay kit; DoGEN, Seoul, Korea). Absorbance was measured at 450 nm (VICTOR3).

### 4.7. Cell-Based ROS Scavenging Assay

Antioxidant activities were evaluated using DCHF-DA (Sigma–Aldrich) as previously described [32]. Caco-2 cells or HaCaT cells were plated on a 96-well black plate at a density of 1 × 10^4^ cells/well in 100 μL MEM and incubated for 24 h. To evaluate oxidative stress triggered by marine algae extracts, the cells were washed with pH 7.4 PBS and incubated overnight with 1 μg/mL marine algae extract in serum-free medium. The antioxidant activity of the extracts was assessed by adding 250 mM H_2_O_2_ either at the same time as the extract or after the cells were first incubated with the extract. The cells were then washed with pH 7.4 PBS and incubated for 30 min with pH 7.4 PBS containing 10 μM DCHF -DA (Sigma–Aldrich). Fluorescence was measured using a spectrofluorometer (VICTOR3) with excitation and emission wavelengths of 485 nm and 535 nm, respectively.

### 4.8. Real-Time Polymerase Chain Reacion (PCR) Analysis

Total RNA was extracted using QIAzol^®^ lysis reagent (Qiagen, Hilden, Germany) and subsequently column-purified with an RNeasy^®^ Mini Kit (Qiagen). The RNA (500 ng) was then treated with DNase I (New England Biolabs, Ipswich, MA, USA), and cDNA was synthesized using an iScript™ cDNA synthesis kit (Bio-Rad Laboratories, Hercules, CA, USA). Real-time PCR was performed using iQ SYBR^®^ Green Supermix (Bio-Rad Laboratories) on a CFX Connect™ real-time PCR detection system (Bio-Rad Laboratories). The primers are listed in Table 1.

### 4.9. TEER Assay

Caco-2 cells were seeded on tissue culture polycarbonate membrane filters (pore size, 0.4 μm) on 24 well transwell plates (SPL, Gyeonggi, Korea) at a seeding density of 5 × 10^4^ cells/cm^2^. Culture medium was added to the upper and lower chambers and changed every second day. The cells were allowed to differentiate for 14 days after seeding. Prior to TEER measurement, the standard medium was substituted with media containing 0.01 μg/mL or 0.1 μg/mL LPS with or without 1 μg/mL Antarctic algae extract. The electrical resistance was measured using a Millicell ERS meter (Millipore, Bedford, MA, USA) and calculated as a percent change of Ω cm^2^ compared with the control group.

### 4.10. FITC-Dextran Permeability Assay

HaCaT cells were seeded on tissue culture polycarbonate membrane filters (pore size, 0.4 μm) on 24 well transwell plates (SPL) at a seeding density of 1.0 × 10^5^ cells/cm^2^. Epithelial permeability across HaCaT monolayers was assessed by measuring the flux of 4-kDa FITC-labeled dextran (2 mg/mL) (Sigma–Aldrich) from the apical chamber to the basolateral chamber of the transwell plate. FITC-dextran was added to the apical chamber and incubated for 2 h at 37 °C. Fluorescence in the basolateral compartment was then measured at an excitation of 485 nm and emission of 535 nm (VICTOR X4, PerkinElmer). 

### 4.11. Statistical Analysis

All experiments were performed in duplicate or triplicate. The sample size for each experiment was based on published literature with a similar methodology. Two-group comparisons were performed with a two-tailed unpaired Student’s t-test. Differences between groups were examined for statistical significance using one-way ANOVA with the Tukey post hoc test. A *p* value < 0.05 was considered statistically significant. GraphPad Prism 9 (GraphPad, San Diego, CA, USA) was used for the data analysis. There were no studies in which the investigators were blinded. 

## 5. Conclusions

This study identified the Antarctic marine algae *H. grandifolius*, *P. cartilagineum*, *P. antarcticus,* and *Kallymenia* as potential sources for therapeutics to promote intestinal and skin epithelial barrier protection. Extracts of these algae contain abundant fatty acids and lipid compounds that contribute to barrier-protective activities and reflect adaptations to optimize the compositional features of the algae for the extreme polar environment. Antarctic marine algae extracts scavenged specific radicals, protected cell viability, scavenged intracellular ROS, and inhibited inflammatory cytokines that had been produced in response to inflammatory or UVB-induced damage. Taken together, our results suggest that Antarctic marine algae are a promising source of compounds that promote epithelial homeostasis of the intestine and skin and protect barrier tissues against inflammatory damage. 

## Figures and Tables

**Table 1:** Compounds identified in *Himantothallus grandifolius* extract.

	Retention Time (min)	Compound	% of Area
1	13.495	n-Hexadecanoic acid	7.278
2	14.806	9-Octadecanoic acid	3.744
3	14.974	Octadecanoic acid	15.841
4	18.674	1,4-Benzenedicarboxylic acid, bis(2-ethylhexyl) ester	2.466
5	20.802	Vitamin E	2.008
6	21.844	Stigmasta-5,24(28)-dien-3-ol, (3.beta.,24Z)-	21.153

**Table 2:** Compounds identified in *Plocamium cartilagineum* extract.

	Retention Time (min)	Compound	% of Area
1	10.220	1,4-Cycloheptadiene, 6-(2-butynyl)-	3.644
2	10.782	2,3-Dimethylanisole	6.45
3	11.054	Benzoic acid, 3-methyl	3.603
4	11.216	Heptadecan	4.415
5	11.630	Benzamide, 3-amino	2.558
6	11.843	Tetradecanoic acid	5.265
7	12.419	Neophytadiene	6.068
8	12.671	4-Pyridinecarboxaldehyde N-oxide	3.611
9	13.493	n-Hexadecanoic acid	38.686
10	14.651	benzenesulfonamide, N-(2,5-dichlorophenyl)-4-methyl	9.51
11	15.919	2-Heneicosanone	2.522
12	20.815	2,2,3,3-Tetrafluoro-5-(1,1,2,2-tetrafluoroethoxy)-2,3-dihydrobenzofuran	3.049
13	21.837	3-(Methylsulfanyl)-4-oxo-4,5,6,7-tetrahydro-2-benzothiophene-1-carboxylic acid, trimethylsilyl ester	4.82

**Table 3:** Compounds identified in *Phaeurus antarcticus* extract.

	Retention Time (min)	Compound	% of Area
1	10.388	1,4-Cycloheptadiene, 6-(2-butynyl)-	3.644
2	11.578	2,3-Dimethylanisole	6.45
3	11.856	Benzoic acid, 3-methyl	3.603
4	12.419	Heptadecan	4.415
5	13.480	n-Hexadecanoic acid	8.876
6	14.030	cis-5,8,11,14,17-Eicosapentaenoic acid	7.56
7	14.573	Phytol	2.747
8	14.722	(6Z,9Z,12Z,15Z)-Methyl octadeca-6,9,12,15-tetraenoate	11.451
9	14.767	9,12-Octadecadienoic acid (Z,Z)-	4.796
10	14.819	9,12,15-Octadecatrienoic acid, (Z,Z,Z)	16.811
11	15.951	Arachidonic acid	2.098
12	15.996	cis-5,8,11,14,17-Eicosapentaenoic acid	15.481
13	19.302	Eicosapentaenoic Acid methyl ester	1.11
14	20.803	Vitamin E	0.548
15	21.838	Stigmasta-5,24(28)-dien-3-ol, (3.beta.,24Z)-	4.126

**Table 4:** Compounds identified in *Kallymenia antarctica* extract.

	Retention Time (min)	Compound	% of Area
1	10.392	Pentadecanal	7.278
2	11.216	Heptadecane	3.744
3	12.44	2-Pentadecanone, 6,10,14-trimethyl	15.841
4	13.48	n-Hexadecanoic acid	39.977
5	14.819	9-Octadecenoic acid	12.005
6	20.815	Cholesterol	21.153

## Data Availability

The datasets generated and/or analyzed during the study are available from the corresponding author upon reasonable request.

## Supplementary Materials

The following supporting information can be downloaded at: https://www.mdpi.com/article/10.3390/md20090562/s1, Figure S1: Optimal stress conditions that decrease survival of Caco-2 and HaCaT cells; Figure S2: The optimal concentration of H_2_O_2_ to increase reactive oxygen species (ROS) levels in Caco-2 cells and HaCaT cells; Figure S3: Optimal inflammatory stimulation and UVB irradiation to induce inflammatory mediators in Caco-2 and HaCaT cells, respectively; Figure S4: LPS treatment conditions to increase permeability in Caco-2 cells and HaCaT cells; Table S1: Primer sequences for real-time PCR.
