# Supplementary material for: Antarctic Marine Algae Extracts as a Potential Natural Resource to Protect Epithelial Barrier Integrity
Source: Mar Drugs. 2022 Aug 31;20(9):562. doi: 10.3390/md20090562 (PMC9503798; doi:10.3390/md20090562)
Supplement: Supplementary file 1 [file marinedrugs-20-00562-s001.zip › marinedrugs-1862910-supplementary.pdf]

Supplementary Information

1. Supplementary Figures and Figure legends

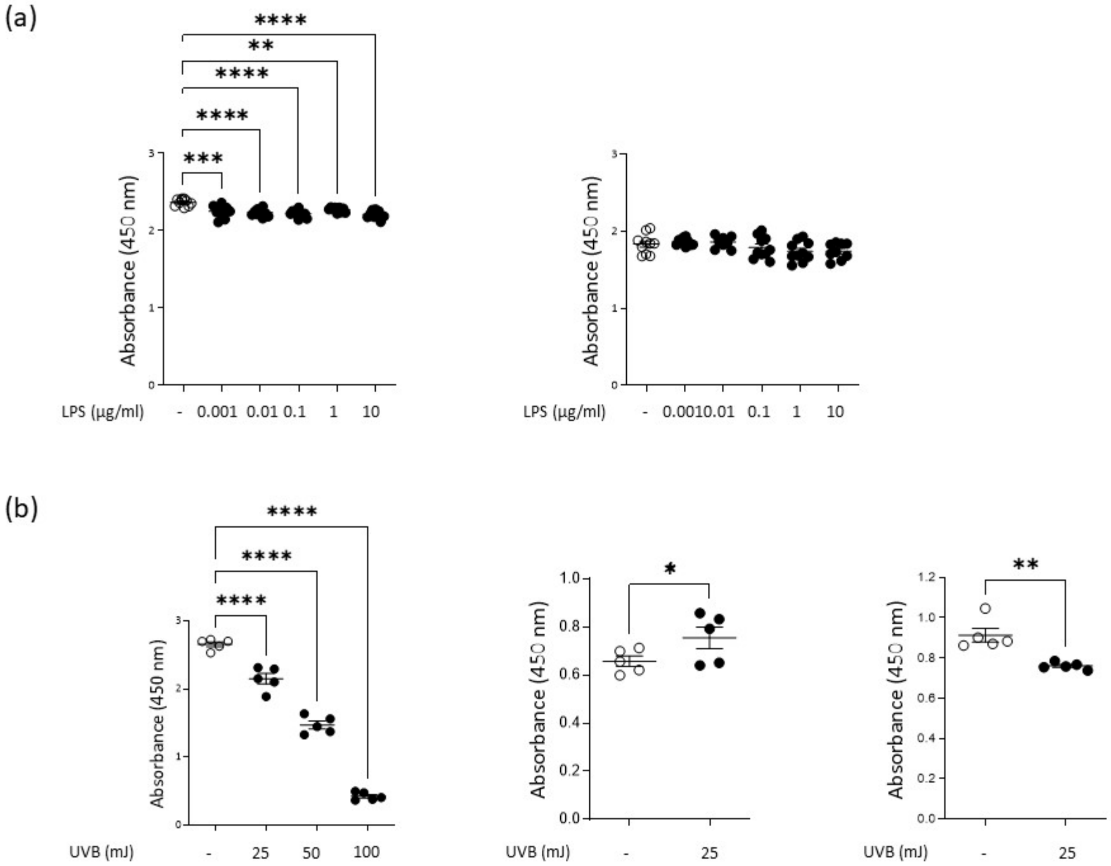

Figure S1. Optimal stress conditions that decrease survival of Caco-2 and HaCaT cells. (a) Caco-2 cell viability with lipopolysaccharide (LPS) treatment after 18 h (left) and 2 h (right). (b) Viability of ultraviolet (UV) B irradiated HaCaT cells after 18 h (left), 2 h (middle), and 4 h (right) of culture. Data are presented as the mean ± SD. *P* values are determined by one-way ANOVA with Turkey's multiple comparisons test. *$P < 0.05$, **$P < 0.01$, and ****$P < 0.0001$.

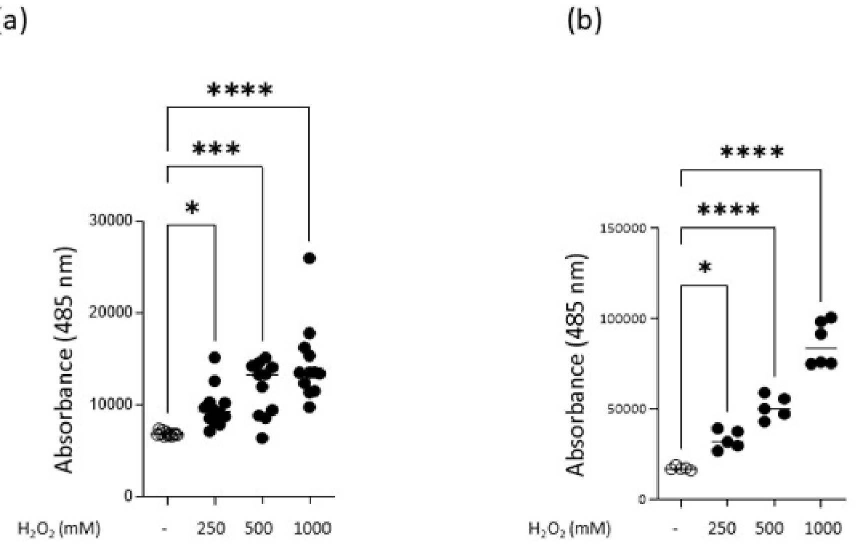

Figure S2. The optimal concentration of H_2O_2 to increase reactive oxygen species (ROS) levels in Caco-2 cells and HaCaT cells. (a and b) Oxidation of Caco-2 cells (a) and HaCaT cells (b) treated with the indicated concentrations of H_2O_2 for 18 h. Data are presented as the mean ± SD. *P* values are determined by one-way ANOVA with Turkey's multiple comparisons test. **P* < 0.05, ****P* < 0.001, and *****P* < 0.0001.

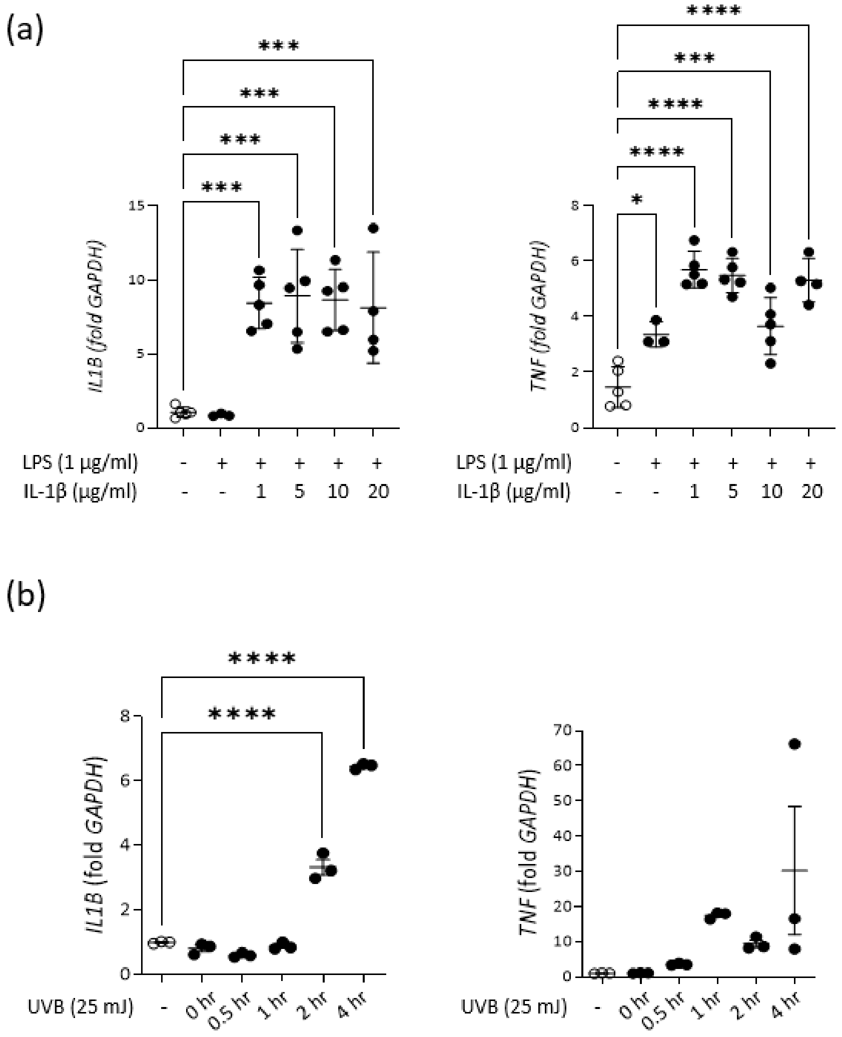

Figure S3. Optimal inflammatory stimulation and UVB irradiation to induce inflammatory mediators in Caco-2 and HaCaT cells, respectively. (a) mRNA expression of *IL1B* and *TNF* in Caco-2 cells stimulated with LPS (1 μg/mL) and various concentrations of IL-1β. (b) mRNA expression of *IL1B* and *TNF* in HaCaT cells irradiated with 25mJ/cm^2 UVB and incubated for the indicated times. Data are presented as the mean ± SD. *P* values are determined by one-way ANOVA with Turkey's multiple comparisons test. ****P* < 0.001 and *****P* < 0.0001.

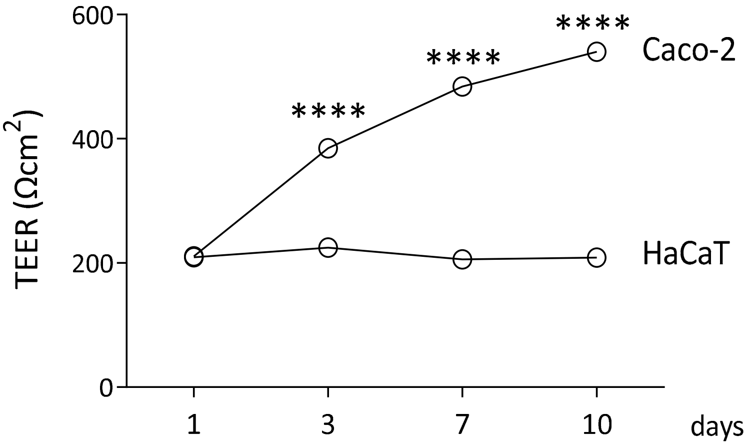

Figure S4. LPS treatment conditions to increase permeability in Caco-2 cells and HaCaT **cells.** Transepithelial electrical resistance (TEER) of polarized Caco-2 cells and monolayer of HaCaT cells. Data are presented as the mean ± SD. *P* values are determined by two-way ANOVA with Bonferroni's multiple comparisons test. ****$P < 0.0001$.

2. Supplementary Table

Table S1. Primer sequences for real-time PCR

Target gene	Primer sequence
S100A8	Forward: 5′- ACCGAGTGTCCTCAGTATATCA -3′<br>Reverse: 5′- CTGGAGAAAGCCTTGAACTCTA -3′
IL1B	Forward: 5′- CCACAGACCTTCCAGGAGAATG -3′<br>Reverse: 5′- GTGCAGTTCAGTGATCGTACAGG -3′
IL8	Forward: 5′- AGAGTGATT GAGAGTGGACC -3′<br>Reverse: 5′- ACTTCTCCACAACCC TCTG -3′
TNF	Forward: 5′- CCTGTGAGGAGGACGAACAT -3′<br>Reverse: 5′- GGTTGAGGGTGTCTGAAGGA -3′
RIPK3	Forward: 5′- GCTGCTGTCTCCACGGTAA -3′<br>Reverse: 5′- CCATCCATTTCTGTCCCTCCTT -3′
GADPH	Forward: 5′-CTGGGCTACACTGAGCACC-3′<br>Reverse: 5′-AAGTGGTCGTTGAGGGCAATG-3′
